# Safety and Immunogenicity of SW-BIC-213, a Modified COVID-19 Lipo-Polyplex mRNA Vaccine, in Laotian Healthy Adults Aged 18 Years and Above: A Phase 1/2 Trial

**DOI:** 10.3390/vaccines14080708

**Published:** 2026-08-18

**Authors:** Ying Yuan, Yichao Zeng, Mingxiong Zhu, Ziyu Sun, Weiwei Xu, Yinran Hu, Guanzhou Liu, Mingyun Shen, Chen Yang, Di Gao

**Affiliations:** 1 Hangzhou Institute for Advanced Study, University of Chinese Academy of Sciences, Hangzhou 310024, China; yuanying25@mails.ucas.ac.cn (Y.Y.); zengyichao25@mails.ucas.ac.cn (Y.Z.); gavincoder@163.com (M.Z.); 15727990279@163.com (W.X.); 2 School of Basic Medical Sciences, Shandong University, Baotuquan Campus, Jinan 250100, China; 3 School of Life Sciences, Nanjing University, Xianlin Campus, Nanjing 210093, China; 4 School of Biomedical Outstanding Engineers, China Pharmaceutical University Jiangning Campus, Nanjing 211198, China; smy@cpu.edu.cn

**Keywords:** SARS-CoV-2, mRNA vaccine, SW-BIC-213, LPP, clinical trial, Laos

## Abstract

Background/Objectives: Messenger RNA vaccines can induce strong immune responses, but delivery, biodistribution, and storage stability remain important considerations. We evaluated the safety and immunogenicity of SW-BIC-213, a Lipo-Polyplex (LPP)-based mRNA vaccine against SARS-CoV-2, in healthy adults in Laos. Methods: We conducted a seamless phase 1/2 clinical trial in healthy adults. Phase 1 enrolled adults aged 18–60 years in an open-label, single-arm dose-escalation study; phase 2 enrolled adults aged ≥18 years in a randomized, double-blind, placebo-controlled study. Participants received two doses 21 days apart. The primary endpoints were safety in phase 1 and safety and immunogenicity in phase 2. Results: In phase 1, 41 participants received 25 or 45 μg of SW-BIC-213. In phase 2, 480 participants were randomized in a 2:2:1 ratio to receive 25 μg, 45 μg, or placebo. All phase 1 adverse reactions were grade 1 or 2; grade 3 reactions in phase 2 were limited to transient fever. At 14 days after the second dose, pseudovirus neutralizing-antibody seroconversion exceeded 99% against wild-type virus, 98% against Delta, 84% against Omicron BA.1, and 88% against BA.2. Neutralizing-antibody titers were higher in both vaccine groups than in the placebo group (*p* < 0.0001). Conclusions: Two doses of SW-BIC-213 showed an acceptable safety profile and substantial humoral immunogenicity in healthy adults aged ≥18 years.

## 1. Background

Since its emergence, COVID-19 has had a substantial global impact [1]. According to the World Health Organization (WHO), more than 767 million confirmed COVID-19 cases and 6.9 million deaths had been reported as of June 2023 [2]. Vaccination remains one of the most effective strategies for reducing transmission and preventing severe disease [3].

In response to the COVID-19 outbreak caused by the SARS-CoV-2 virus, numerous vaccine development programs have been launched. As of March 2023, the World Health Organization’s vaccine tracker reported a total of 382 vaccine candidates under development [4].

These candidates use various platforms, including live attenuated vaccines, recombinant protein vaccines, viral-vector vaccines, DNA vaccines, and RNA vaccines [5,6,7,8,9,10]. Among these platforms, mRNA vaccines have demonstrated high protective efficacy [5,6,7]. BNT162b2 and mRNA-1273 are representative mRNA vaccines against SARS-CoV-2. BNT162b2, administered as a two-dose regimen of 30 μg per dose with a 21-day interval, showed a favorable safety profile and 95% efficacy against COVID-19 [11]. Similarly, mRNA-1273 was evaluated in its pivotal efficacy trial as a two-dose regimen of 100 μg per dose with a 28-day interval and showed 94.1% efficacy in preventing COVID-19, including severe disease [12]. These vaccines made major contributions to pandemic control and demonstrated the feasibility of mRNA vaccine technology.

For various reasons, these two mRNA vaccines have not been introduced into mainland China. One possible reason is that they have been associated with more frequent reactogenicity than inactivated COVID-19 vaccines widely used in China [13,14]. Nevertheless, the rapid manufacturing process and strong immunogenicity of mRNA vaccines have encouraged several Chinese companies, including StemiRNA Therapeutics, to develop domestic COVID-19 mRNA vaccines.

A variety of delivery systems have been developed for mRNA vaccines, but concerns remain regarding unfavorable biodistribution and the relative instability of mRNA vaccines [12,15,16,17]. To address these issues, we developed a core–shell structured Lipo-Polyplex (LPP) nanoparticle for mRNA delivery. In contrast to conventional lipid nanoparticles (LNPs), in which ionizable lipids are the principal component responsible for mRNA encapsulation, the LPP platform first condenses mRNA with positively charged protamine sulfate to form a compact core, which is subsequently coated with a lipid shell. This core–shell organization is designed to improve mRNA protection, lymphoid-organ biodistribution, and storage stability. In preclinical studies, LPP was mainly distributed in lymph nodes and the spleen and could be stored at −20 °C for long-term storage. The LPP platform has been used to deliver our first-generation COVID-19 mRNA vaccine SW0123 [18], which was highly immunogenic in animal models.

SW-BIC-213 is our next-generation investigational mRNA vaccine encoding a modified full-length spike protein based on the ancestral Wuhan-Hu-1 isolate of SARS-CoV-2. The Wuhan-Hu-1 spike sequence was selected because it is the original reference sequence for SARS-CoV-2, has been widely used as a standard antigen backbone in early COVID-19 vaccine development, and allows comparison with previously reported prototype-strain assays. The encoded spike includes SARS-CoV-2-specific stabilizing mutations, including K986P/V987P (2P) substitutions to maintain the prefusion spike conformation, a 682-QSAQ-685 substitution to disrupt the furin cleavage site and reduce S1/S2 cleavage, and D614G, a spike mutation associated with enhanced spike expression and a dominant early pandemic variant [19,20]. Although the current epidemiological context includes widespread vaccine-induced, infection-induced, or hybrid immunity, evaluation of a primary two-dose regimen remains informative for defining the safety, dose selection, and baseline immunogenicity of this LPP-based platform and for supporting subsequent booster evaluation in previously immunized populations. SW-BIC-213 is also based on the LPP platform [18], and this investigational COVID-19 vaccine was safe and well tolerated in an investigator-initiated trial conducted in China [19]. In this study, we present clinical trial data from a phase 1/2 trial among healthy volunteers in Laos (Lao PDR) to assess the safety and immunogenicity of the LPP-based mRNA vaccine against COVID-19.

## 2. Methods

### 2.1. Trial Design

We conducted a seamless phase 1/2 study in Laos to evaluate the safety and immunogenicity of SW-BIC-213. The phase 1 trial used an open-label, single-arm, dose-escalation design and included healthy individuals aged 18–60 years. The phase 2 trial used a randomized, double-blind, placebo-controlled design and enrolled healthy individuals aged 18 years and older.

The trial received approval from the Ethics Committee and Food & Drug Department of the Ministry of Health, Lao PDR (7745). The protocol and each amendment underwent review by the Independent Ethics Committee of the Lao People’s Democratic Republic Ministry of Health National Ethics Committee for Health Research. The study adhered to the principles outlined in the Declaration of Helsinki and Good Clinical Practice Guidelines. Safety assessment, including the necessity of study suspension or termination, was conducted by an independent Data Safety Monitoring Board (DSMB).

This phase 1 and phase 2 trial was registered with the identifier NCT05144139 on ClinicalTrials.gov.

### 2.2. Participants

Participants in both the phase 1 and phase 2 trials were recruited from Savannakhet Provincial Hospital and Champhone District Hospital, two healthcare facilities in southern Savannakhet province, Laos. The inclusion and exclusion criteria were the same for phase 1 and phase 2. In general, participants were in overall good health, as established by medical history, physical examination, and laboratory tests at the screening visit. Both male and female participants were included and agreed to use contraception during the trial. Pregnant participants were excluded. Participants with a known history of SARS-CoV-2, SARS-CoV-1, or MERS-CoV infection were excluded. Known previous infection was screened by medical history, self-reported infection history, interview, physical examination, and review of COVID-19-related symptoms within 14 days before enrollment, including fever, dry cough, fatigue, nasal congestion, runny nose, sore throat, myalgia, diarrhea, shortness of breath, and dyspnea. Baseline serum samples were collected before vaccination for immunogenicity assessment; baseline serology was used to interpret antibody responses rather than as the sole criterion for excluding asymptomatic previous infection. Participants with serious cardiovascular disease, hypertension, acute respiratory disease, or kidney disease were excluded. A detailed list of the inclusion and exclusion criteria is provided in File S1A. The full clinical trial protocol is provided in File S2.

### 2.3. Interventions

The SW-BIC-213 vaccine was composed of an mRNA encoding the full-length spike glycoprotein of the Wuhan-Hu-1 isolate of SARS-CoV-2. This mRNA included artificial mutations, including the K986P/V987P (2P) prefusion stabilizing mutations, the 682-QSAQ-685 substitution at the furin cleavage site, and the D614G mutation. The vaccine was formulated with LPP and was developed and manufactured by StemiRNA Therapeutics Co., Ltd., in Shanghai, China, in accordance with good manufacturing practice guidelines. The 25 μg and 45 μg dose levels were protocol-specified clinical dose levels selected to evaluate a lower and higher dose while maintaining a margin below the nominal 50 μg vial content for practical dose withdrawal and administration. The vial fill volume and extractable volume were controlled according to manufacturing release specifications. Briefly, purified mRNA was diluted in aqueous buffer and first complexed with a cationic component to form the mRNA-containing core. The aqueous phase was then combined with the lipid mixture in ethanol at a 3:1 volume ratio (aqueous:ethanol) using a microfluidic mixer (Inano D, Micro&Nano Technology Inc., Shanghai, China), followed by buffer exchange, sterile filtration, and aseptic filling. Batch release testing included appearance, identity, RNA content, encapsulation-related quality attributes, particle size, polydispersity, pH, sterility, bacterial endotoxin, and potency-related testing according to the manufacturer’s quality control specifications. The vaccine was supplied as a buffered liquid solution in vials, with each vial nominally containing 50 μg of the vaccine in 0.5 mL. It was stored at temperatures ranging from −25 °C to −15 °C until use.

Before administration, frozen vaccine vials were thawed under controlled conditions according to the protocol and the manufacturer’s handling instructions, kept at 2–8 °C after thawing, and prepared for administration during the same vaccination session. The placebo consisted of a commercial, pre-packaged, preservative-free saline solution containing 0.9% NaCl. Each participant received two doses of SW-BIC-213 vaccine candidate, at either 25 μg or 45 μg per dose, or placebo. The injected active vaccine volumes were 0.25 mL for the 25 μg dose and 0.45 mL for the 45 μg dose; within each sequential dose cohort, the saline placebo was prepared to match the corresponding active-dose injection volume. Vaccinations were administered in the upper-arm deltoid muscle, with an interval of 21 days between doses. Following each dose, participants were observed on-site for 30 min, during which any adverse reactions were recorded by the investigators. Body temperature was measured consistently at the axillary site using a thermometer according to the site standard operating procedure. Participants documented body temperature and adverse events on daily cards during the first 7 days after each vaccination. Participants reported any adverse events occurring within days 7–21 and days 7–28 after the first and second vaccination, respectively, using contact cards. Any serious adverse events or pregnancies that developed from the first vaccination until the end of the study were monitored, and follow-up was ongoing.

In phase 1 only, participants underwent blood and urine sample collection before and 4 days after each dose for hematology and chemistry laboratory tests to assess any toxicity following vaccination. The enrollment of each dosage group in the phase 2 study was carried out only if the corresponding dosage group in the phase 1 study demonstrated a favorable safety profile.

### 2.4. Safety Assessment

Solicited local adverse events at the injection site within 7 days after vaccination included pain, pruritus, induration, swelling, and redness. Solicited systemic adverse events within 7 days after vaccination included fever, fatigue, headache, myalgia, chills, arthralgia, nausea, swollen lymph nodes (lymphadenopathy), diarrhea, vomiting, and acute allergic reaction. Unsolicited adverse events occurring within 0–21 days and 0–28 days after the first and second dose, respectively, were collected. Unsolicited adverse events were adverse events that were not included in the protocol-defined solicited adverse reactions. The severity of adverse events was assessed according to the Division of AIDS (DAIDS) Table for Grading the Severity of Adult and Pediatric Adverse Events (Version 2.1) [21]. The severity of abnormal vital signs and laboratory test results was primarily evaluated using the Guidelines for Adverse Event Classification Standards for Clinical Trials of Preventive Vaccines issued by the National Medical Products Administration (NMPA) in 2019 [22], and further complemented by the United States Food and Drug Administration (US FDA) Toxicity Grading Scale for Healthy Adult and Adolescent Volunteers Enrolled in Preventive Vaccine Clinical Trials [23]. The causal association between adverse events and vaccination was finally determined by experienced physicians based on clinical judgment.

### 2.5. Immunogenicity Assessment

Blood samples were collected from participants before each dose of vaccination and 14, 90, and 180 days after the second dose. These samples were used to evaluate antibody responses, specifically Spike receptor binding domain (RBD) specific IgG antibodies and neutralizing antibodies against SARS-CoV-2. The detailed protocols [19,24] were included in File S1A.

### 2.6. Outcomes

In the phase 1 trial, the primary outcome focused on the safety of the COVID-19 mRNA vaccine in healthy people aged 18–60 years. The secondary outcome assessed the humoral immunogenicity and long-term safety for six months.

In the phase 2 trial, the primary outcome aimed to evaluate the humoral immunogenicity and safety in healthy people aged 18 years and above. The secondary outcome examined the long-term safety and persistence of humoral immunity for six months.

Safety outcomes included the incidence of local/systemic solicited adverse reactions/events (0–6 days after each vaccination dose), unsolicited adverse events (0–21 days and 0–28 days after the first and second dose of immunization, respectively), medically attended adverse events, adverse events of special interest (AESI), and serious adverse events from the first dose of vaccination to 28 days after completing the full course of immunization.

Immunogenicity outcomes included seroconversion rate and geometric mean titer (GMT) of SARS-CoV-2 spike protein-specific IgG antibodies and neutralizing antibodies 14 days after the second dose. Neutralizing antibodies were evaluated using pseudovirus neutralization assays against the wild-type (WT), Delta, Omicron BA.1, and Omicron BA.2 strains, and using a live-virus neutralization assay against the WT strain. In addition, RBD-specific IgG antibodies were investigated in the phase 1 trial as an exploratory endpoint.

### 2.7. Sample Size

The superiority hypothesis between treatment and control groups was tested. H0: p1 ≤ p2; H1: p1 > p2, where p1 was the seroconversion rate of the vaccine group and p2 was the seroconversion rate of the placebo group. The superiority hypothesis was tested at a one-sided significance level of 2.5%.

In the phase 1 trial, the probability of observing a particular adverse event with an incidence of 8% at least once in 20 participants in each dose group was 81.1%. The actual enrolled sample size was 41. In the phase 2 trial, the sample size calculation was based on the assumption that the seroconversion rates of the vaccine and placebo groups would be 80% and 10%, respectively, and the test level would be a unilateral α = 0.025. We calculated that, with a sample size of 144 subjects in each vaccine group and 36 subjects in each placebo group for adults and 48 subjects in each vaccine group and 12 subjects in each placebo group for seniors, the difference between the vaccine and placebo could be estimated with at least 99% power, including the consideration of a 33% dropout rate. The actual enrolled sample size was 480.

### 2.8. Randomization and Blinding

The phase 1 trial was open-label, and participants were enrolled in the order of recruitment. Therefore, randomization and blinding were not used in phase 1. Eligible participants were assigned in a 1:1 ratio to receive either 25 μg or 45 μg of SW-BIC-213 according to enrollment sequence. Enrollment proceeded stepwise from the 25 μg dose group to the 45 μg dose group; enrollment into the 45 μg group started only after a preliminary 7-day safety assessment of the 25 μg group was favorable.

The phase 2 trial used a randomized, blinded, placebo-controlled parallel design. Participants were enrolled into two age cohorts, adults (≥18 to ≤60 years) and seniors (>60 years), at an approximate 3:1 ratio. Within each dose cohort, participants were randomly assigned at a 4:1 ratio to receive SW-BIC-213 or placebo. Randomization was performed using an interactive response technology (IRT) system. A randomization statistician generated the participant randomization list using block randomization (block size 30) in SAS version 9.4, and the list was imported into the IRT system for treatment allocation. Authorized unblinded pharmacists prepared the vaccine or placebo according to the IRT allocation, and vaccination staff administered the investigational products. The 25 μg and 45 μg dose cohorts used different active-dose injection volumes, and the placebo volume was matched within each corresponding dose cohort. Participants, investigators involved in safety assessment, laboratory staff, and the sponsor remained blinded throughout the trial. Unblinded staff had no further involvement in trial assessment and were prohibited from disclosing allocation information. A formal blinding-index or allocation-guess assessment was not collected.

Details of the blinding procedures are provided in File S1B.

### 2.9. Statistical Analysis

The safety analysis included all participants who received at least one dose of the investigational vaccine. Immunogenicity analyses were primarily based on the per-protocol set (PPS) and supplemented by the full analysis set (FAS) at various time points, as defined in the study protocol. The FAS comprised all participants who received at least one dose of vaccination and had valid pre-vaccination antibody results; participants who received an incorrect intervention were analyzed for immunogenicity according to the randomized group, following the intention-to-treat principle. The PPS included participants who were enrolled according to the inclusion and exclusion criteria, completed the vaccination course as defined in the protocol, and had valid pre- and post-vaccination immunogenicity data. Safety endpoints were presented as the percentage of participants experiencing adverse events or serious adverse events during the observation period. Fisher’s exact test was used to compare differences in proportions across groups. Immunological analyses included seroconversion rates and GMTs at specified time points. We calculated 95% confidence intervals using the Clopper-Pearson method. Chi-square or Fisher’s exact tests were used to compare seroconversion rates among groups. GMTs were compared using Student’s *t*-test after log transformation. When ANOVA indicated an overall difference among three or more groups, prespecified pairwise comparisons were performed. No formal multiplicity adjustment, such as Bonferroni correction, was applied; therefore, *p* values from multiple comparisons should be interpreted as nominal and exploratory. All statistical analyses were conducted using SAS version 9.4.

## 3. Results

### 3.1. Patient Characteristics

**Figure 1:**
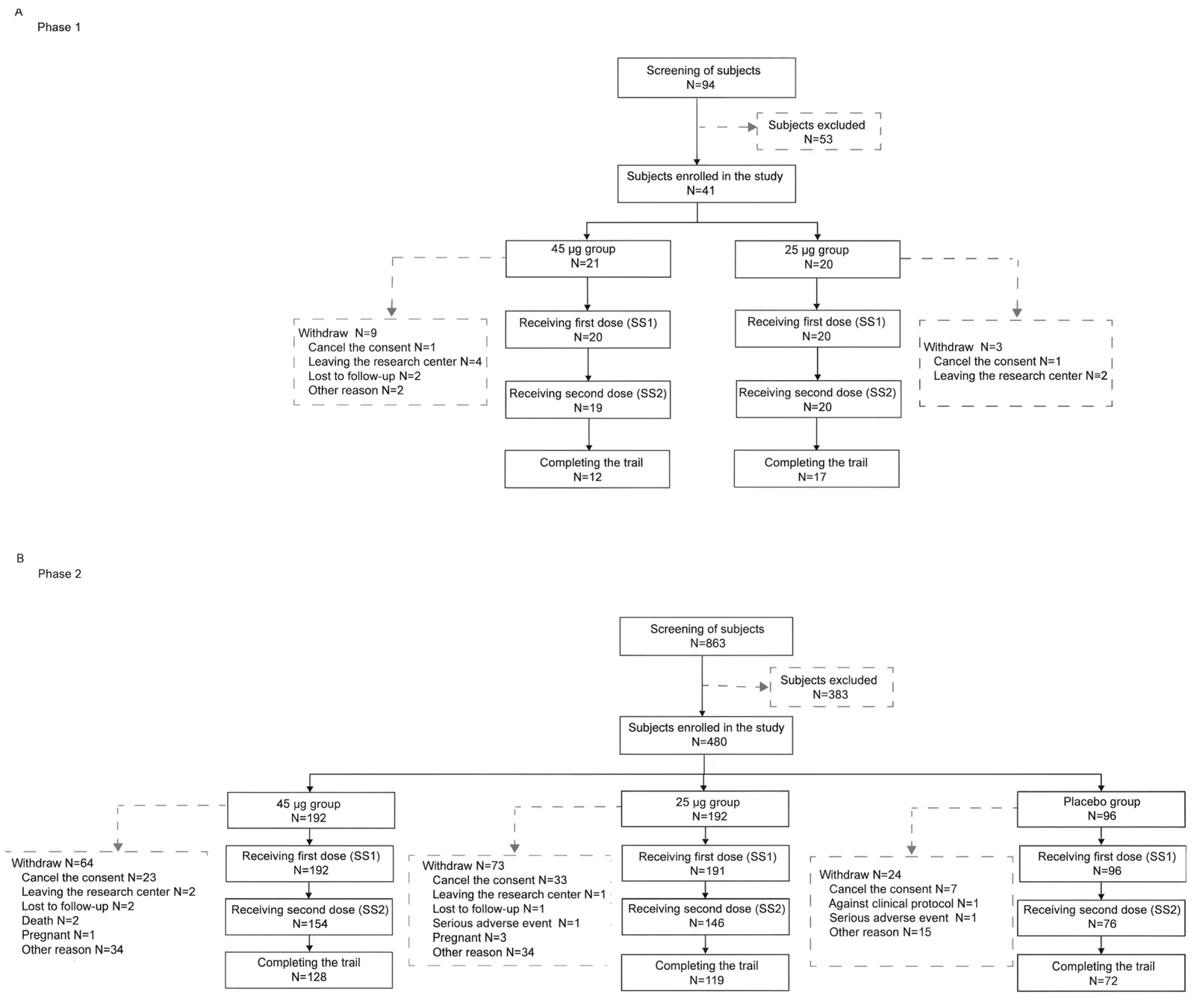
Screening and randomization of participants. The screening and randomization profiles of phase 1 (**A**) and phase 2 (**B**) trials are shown.

Between 3 December 2021, and 31 March 2022, a total of 94 individuals underwent recruitment and screening for the phase 1 trial in Vientiane and Savannakhet, Laos (Figure 1A). After excluding 53 individuals, 41 eligible participants were assigned to receive either the 25 μg or 45 μg dose of the vaccine. Of these, 40 participants received the first vaccination and 39 completed the two-dose immunization schedule. Baseline age and other demographic characteristics were balanced between the two vaccination groups (Table 1).

Between 20 January 2022, and 6 July 2022, a total of 863 individuals underwent recruitment and screening for the phase 2 trial in Vientiane and Savannakhet, Laos (Figure 1B). After excluding 383 individuals, 480 eligible participants were randomly assigned to receive the 25 μg dose, the 45 μg dose, or placebo. Baseline age was comparable across groups (Table 1). Other baseline characteristics were generally balanced, except for sex distribution, mainly due to differences in the senior cohort. In the phase 2 study, 479 eligible participants received the first vaccination in the deltoid muscle, and 376 received the second vaccination using the same route and assigned dosage as the first vaccination.

### 3.2. Safety

**Figure 2:**
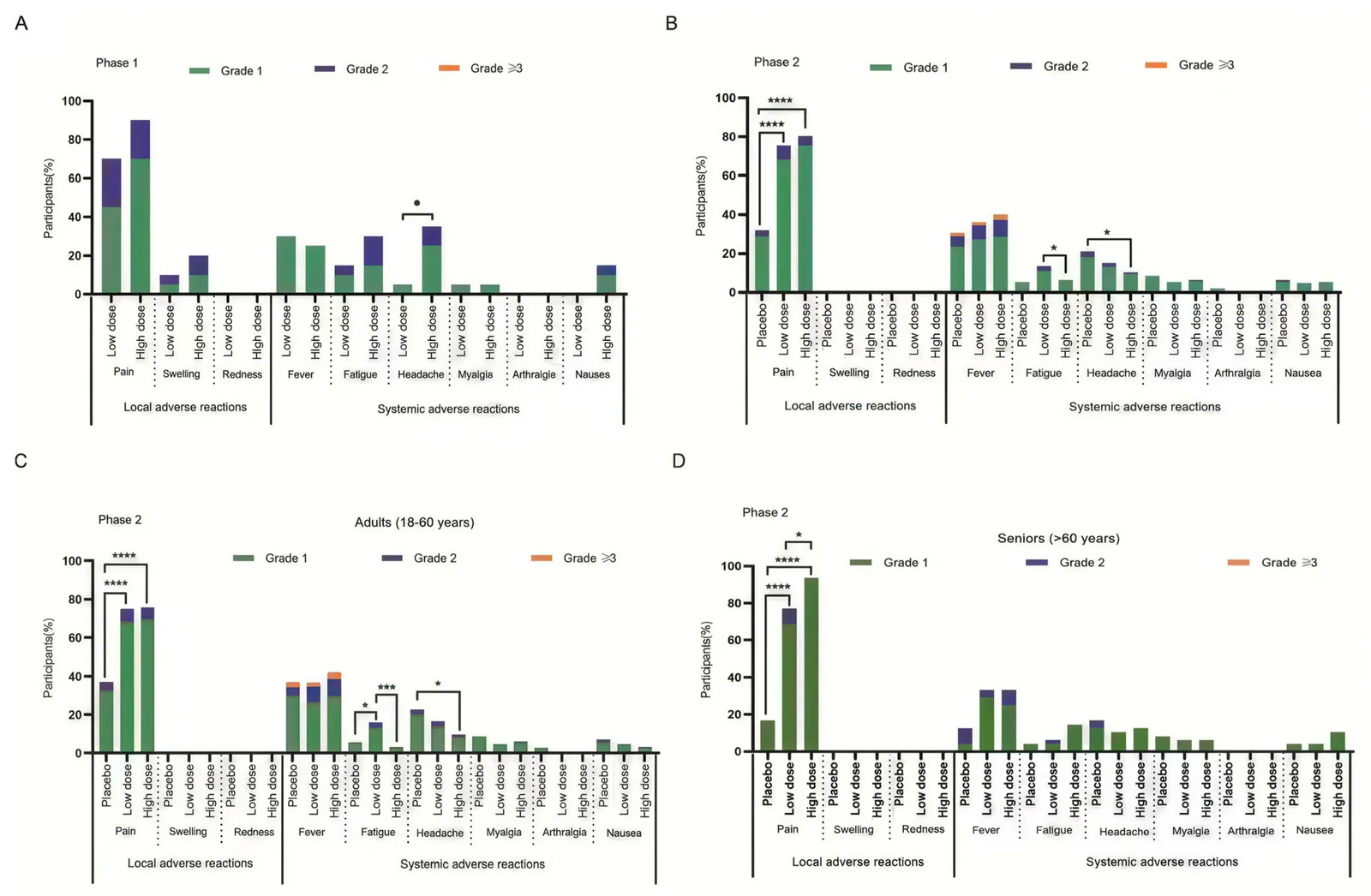
Vaccination-related adverse events and reactions in the phase 1 and phase 2 trials. (**A**) Severity of solicited adverse reactions in the phase 1 trial. (**B**) Severity of solicited adverse reactions in the phase 2 trial. (**C**) Severity of solicited adverse reactions in adults aged 18–60 years in the phase 2 trial. (**D**) Severity of solicited adverse reactions in seniors aged >60 years in the phase 2 trial. Statistical significance is indicated by * (*p* ≤ 0.05), *** (*p* ≤ 0.001), and **** (*p* ≤ 0.0001).

All grades of adverse reactions occurring in a participant were recorded and included in the analysis (Figure 2), rather than only the most severe reaction. The overall adverse reaction rate was defined as the proportion of participants with any adverse reaction; therefore, rates by severity grade do not add up to the overall adverse reaction rate.

In the phase 1 study, the most common adverse reactions were injection-site pain, fever, fatigue, swelling, and headache (Figure 2A). These reactions were limited to grade 1 or 2, and no grade 3 or higher adverse reactions were reported before the data cutoff date. Detailed group-specific percentages are shown in Figure 2A.

Similarly, in the phase 2 study, injection-site pain was the most frequently reported local reaction, while fever, fatigue, and headache were the most common systemic reactions (Figure 2C,D). Most reactions were grade 1 or 2. Grade 3 or higher reactions were limited to fever and occurred at low frequency in adults across both vaccine and placebo groups. No safety signal suggestive of AESI, vaccine-enhanced disease, or other unexpected serious toxicity was identified. Detailed percentages by dose group and age cohort are provided in Figure 2C,D.

Notably, in both the adult and senior cohorts, injection-site pain was significantly more frequent in the 45 μg and 25 μg vaccine groups than in the placebo group (*p* < 0.0001; Figure 2C,D). The incidence of headache in the adult placebo group was slightly higher than that in the 45 μg group (Figure 2C), which may have been related to individual background factors.

Moreover, in the adult cohort, fatigue was more frequent in the 25 μg dose group than in the 45 μg dose group and placebo group (Figure 2C). Other adverse effects did not differ significantly between the two vaccine dose groups. As in phase 1, all adverse symptoms resolved, and most were transient and self-limiting.

Overall, SW-BIC-213 demonstrated a favorable safety profile and was well tolerated by eligible participants aged ≥18 years, regardless of dose or study phase. Local reactions were more common than systemic reactions. The adverse reactions observed during the trial were generally tolerable, transient, and self-resolving. All grade 3 adverse reactions were fever events and were successfully managed.

### 3.3. Immunogenicity Assessments

**Figure 3:**
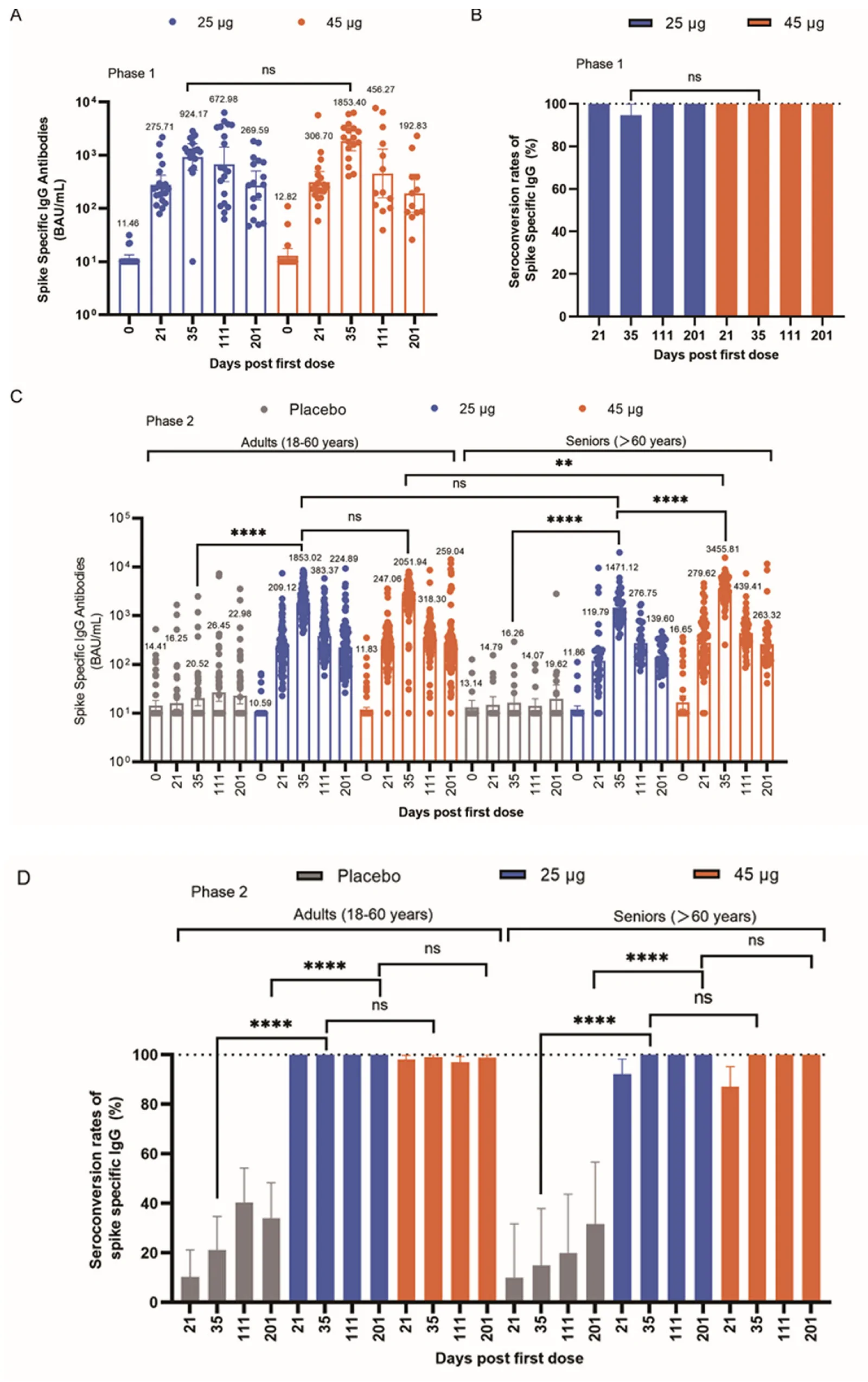
SW-BIC-213 induced spike protein binding antibodies in phase 1 and phase 2 trials. (**A**) Geometric mean SARS-CoV-2 spike protein binding antibody titers determined by ELISA in the phase 1 trial. (**B**) Seroconversion rates in the phase 1 trial. (**C**) Geometric mean spike protein binding antibody titers in the phase 2 trial. (**D**) Seroconversion rates in the phase 2 trial. Statistical significance is indicated by ns (*p* > 0.05), ** (*p* ≤ 0.01), and **** (*p* ≤ 0.0001).

In the phase 1 trial, spike-binding IgG titers measured by ELISA increased from baseline after the first dose and increased further by 14 days after the second dose in both vaccine groups (Figure 3A). Although titers tended to be higher in the 45 μg group than in the 25 μg group after the second dose, the between-group difference was not statistically significant. Seroconversion rates were high after the first dose and remained high through 180 days after full immunization (Figure 3B). Detailed GMTs and seroconversion rates are presented in Figure 3A,B.

Given the importance of the RBD domain of the S protein for viral entry, antibodies targeting this domain were expected to be neutralizing and potentially protective. RBD-binding IgG antibodies showed a pattern similar to that of spike-binding IgG antibodies, increasing after the first dose and rising further after the second dose in both vaccine groups (Figure S1A). Seroconversion rates remained high through 180 days after full immunization, with no statistically significant between-group differences (Figure S1B).

In the phase 2 trial, adults and seniors in both vaccine dose groups showed increased GMTs of S-protein-specific antibodies from baseline to after the first dose and further to 14 days after the second dose (Figure 3C). Antibody titers in both vaccine groups were significantly higher than those in the placebo group in both age cohorts (*p* < 0.0001). Among seniors, the 45 μg dose induced higher titers than the 25 μg dose (*p* < 0.0001). Detailed GMTs are shown in Figure 3C.

Among adults who received SW-BIC-213, seroconversion rates were already high after the first dose and remained high after the second dose (Figure 3D). After full immunization, seroconversion rates in the vaccine groups were significantly higher than those in the placebo group (*p* < 0.0001), with no significant difference between the 45 μg and 25 μg dose groups. Seroconversion rates remained at high levels at 90 and 180 days after full immunization (Figure 3D).

**Figure 4:**
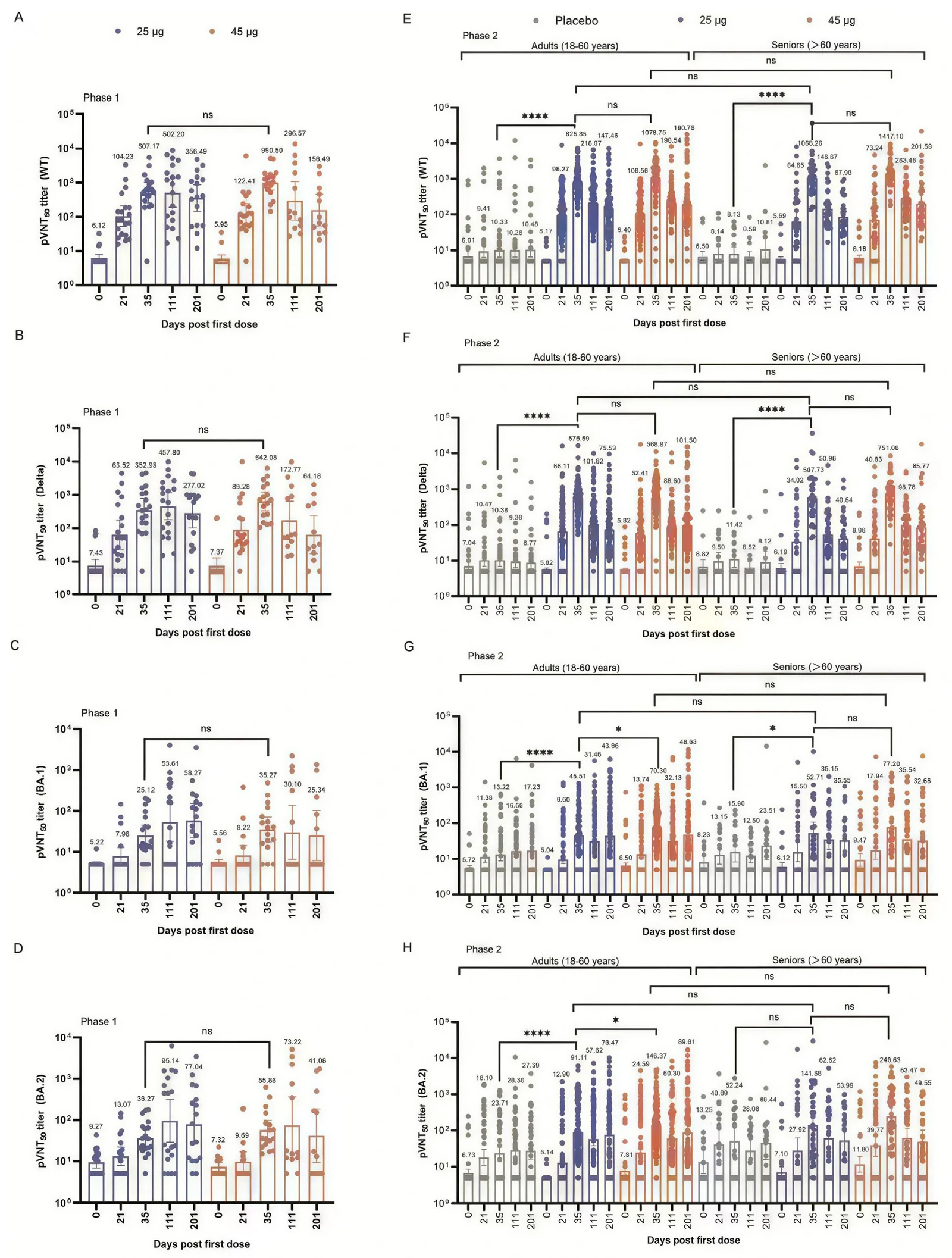
SW-BIC-213 induced pseudovirus neutralizing antibodies in phase 1 and phase 2 trials. (**A**–**D**) Geometric mean titers in the phase 1 trial against prototype/WT, Delta, Omicron BA.1, and Omicron BA.2 strains, respectively. (**E**–**H**) Geometric mean titers in the phase 2 trial against prototype/WT, Delta, Omicron BA.1, and Omicron BA.2 strains, respectively. In phase 2 panels, adult and senior cohorts and the placebo, 25 μg, and 45 μg groups are indicated by the axis labels and figure legends. Statistical significance is indicated by ns (*p* > 0.05), * (*p* ≤ 0.05), and **** (*p* ≤ 0.0001).

Serum samples were also assessed for pseudovirus neutralizing titers against the SARS-CoV-2 prototype strain and three variants of concern, Delta, Omicron BA.1, and Omicron BA.2. In the phase 1 trial, neutralizing GMTs against WT and Delta were higher than those against Omicron BA.1 and BA.2 at 14 days after the second dose (Figure 4A–D). GMTs were consistently higher in the 45 μg group than in the 25 μg group, although the between-group differences were not statistically significant. Seroconversion rates were high for WT, Delta, and BA.1, while the response to BA.2 was lower in the 25 μg group (Figure S2A–D). Detailed values are provided in Figure 4 and Figure S2.

In the phase 2 trial, both vaccine groups showed significantly increased pseudovirus neutralizing antibodies against WT, Delta, BA.1, and BA.2 compared with the placebo group (Figure 4E–H). Responses to Omicron BA.1 and BA.2 were lower than those to WT and Delta, consistent with the immune-evasive properties of Omicron variants. Overall, GMTs were similar or higher in the 45 μg group than in the 25 μg group, and responses in seniors were generally comparable to those in adults. Seroconversion rates remained stable from 90 to 180 days after full immunization, although minor increases at some time points may reflect changes in participant numbers or intercurrent infection during the epidemic. Detailed numerical results are shown in Figure 4 and Figure S2.

**Figure 5:**
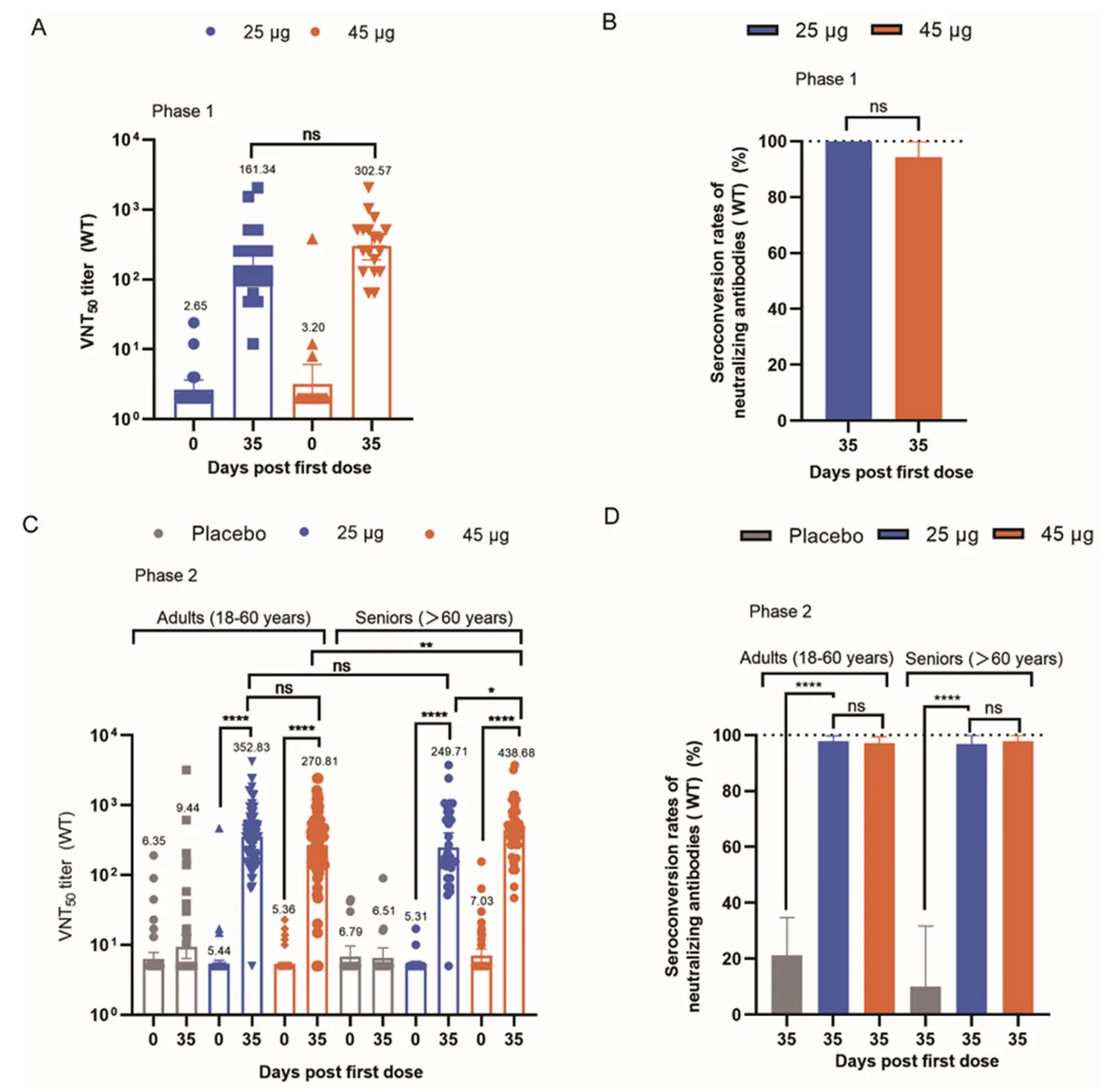
SW-BIC-213 induced neutralizing antibodies in phase 1 and phase 2 trials. (**A**) Geometric mean neutralizing antibody titers determined by microneutralization assay in the phase 1 trial. (**B**) Seroconversion rates in the phase 1 trial. (**C**) Geometric mean neutralizing antibody titers in the phase 2 trial. (**D**) Seroconversion rates in the phase 2 trial. Statistical significance is indicated by ns (*p* > 0.05), * (*p* ≤ 0.05), ** (*p* ≤ 0.01), and **** (*p* ≤ 0.0001).

Live-virus neutralization assays showed that SW-BIC-213 induced neutralizing antibodies against the WT strain in both phases (Figure 5). In phase 1, neutralizing GMTs increased after vaccination and did not differ significantly between the two dose groups. In phase 2, both vaccine groups had significantly higher neutralizing antibody levels than the placebo group in adults and seniors (*p* < 0.0001). Among seniors, the 45 μg dose induced higher titers than the 25 μg dose. Seroconversion rates after the second dose were high in both vaccine dose groups and were significantly higher than those in the placebo group (Figure 5D). Detailed group-specific values are presented in Figure 5.

## 4. Discussion

In both phase 1 and phase 2 trials, two doses of SW-BIC-213 at 25 or 45 μg were well tolerated, with adverse reactions mainly being transient and resolving spontaneously. Notably, no vaccination-related serious adverse event (SAE) or adverse event of special interest, such as antibody-dependent enhancement (ADE) or vaccine-enhanced disease (VED), was observed after the first dose. Adverse reactions in both phases were primarily local solicited reactions, with injection-site pain being the most common symptom. The frequency of solicited systemic adverse reactions was similar between the vaccine and placebo groups in phase 2. Overall, SW-BIC-213 showed a favorable safety profile with generally mild or moderate reactogenicity, comparable to the representative mRNA vaccines mRNA-1273 [12,25] and BNT162b2 [16,26].

Humoral responses have been considered immune correlates of protection against SARS-CoV-2 [27]. SW-BIC-213 induced measurable humoral responses rapidly. For S-protein IgG antibodies, one dose induced a seroconversion rate comparable to that achieved after full immunization, and responses were broadly similar between the 45 μg and 25 μg dose groups. In contrast, pseudovirus neutralizing antibody seroconversion increased with completion of the two-dose regimen. GMT analyses suggested a dose-related increase in IgG and neutralizing antibody responses. Because emerging variants continue to affect vaccine effectiveness, neutralizing antibodies against multiple variants of concern were evaluated. SW-BIC-213 showed responses against Delta relative to WT, while neutralization against Omicron BA.1 and BA.2 was markedly lower, consistent with previous reports of Omicron immune evasion [28,29,30,31,32]. Among participants aged 60 years and older, humoral immunogenicity was comparable to that in adults aged 18–60 years, with a similar safety profile; this is clinically relevant because older adults are at higher risk of severe COVID-19 [33].

The long-term tolerability profile of SW-BIC-213 and persistence of the elicited immune responses were also investigated according to the study protocol. Phase 1 and phase 2 results indicated that seroconversion rates for WT neutralizing antibodies, Delta neutralizing antibodies, and S-protein-specific antibodies remained relatively high at 90 and 180 days after full immunization. Increases in Omicron BA.1 or BA.2 seroconversion rates from 90 to 180 days after full immunization, as well as seroconversion in the placebo group, suggested possible exposure to SARS-CoV-2 during the trial. Because community transmission occurred in Laos during the study, such exposure was likely to occur across groups and should be considered when interpreting persistence data. As SARS-CoV-2 continues to evolve and vaccine-induced protection wanes, many people have experienced breakthrough infections. Hybrid immunity after both vaccination and infection can produce stronger immune responses than vaccination or infection alone, and several studies suggest that hybrid immunity confers broader cross-variant neutralization and more durable protection against new infections [34,35].

This study had several limitations. First, data interpretation was based on a relatively small sample size, and additional data from phase 3 trials are needed to comprehensively evaluate the safety and efficacy of SW-BIC-213 as a booster in heterologous boost regimens. Second, the study population was not ethnically diverse, as all participants were of Asian descent. Third, neutralizing potency against currently circulating Omicron BA.4/5 and later variants was not evaluated because variants of concern were shifting during the study period. Fourth, immunogenicity assessment in this trial was limited to humoral endpoints, including binding IgG and pseudovirus or live-virus neutralization assays. Cellular immune responses were not measured; therefore, the contribution of T-cell immunity to cross-variant protection could not be assessed and should be investigated in future studies. Fifth, although participants with known previous SARS-CoV-2 infection or recent COVID-19-like symptoms were excluded, anti-nucleocapsid antibody testing, systematic PCR screening, and formal baseline seropositivity exclusion were not performed; therefore, asymptomatic or undocumented previous infection and hybrid immunity could not be fully ruled out. Sixth, systematic surveillance data for breakthrough infection during follow-up, including PCR-confirmed infection or serological evidence of intercurrent infection, were not collected; therefore, the persistence analyses should be interpreted cautiously. Seventh, a formal blinding-index or allocation-guess assessment was not collected, and this should be considered when interpreting solicited reactogenicity outcomes. Finally, this study did not include a clinical efficacy endpoint, and no formal correlate of protection was established in this trial; therefore, immunogenicity findings should not be interpreted as direct evidence of clinical protection.

In conclusion, SW-BIC-213 showed an acceptable safety profile and induced measurable humoral immune responses in eligible participants aged ≥18 years in both the phase 1 and phase 2 trials. These findings support further investigation of SW-BIC-213 for COVID-19 vaccination strategies. Because COVID-19 vaccines have been widely used worldwide as primary series, many target populations may already have some degree of pre-existing protection. Therefore, a dose of 25 μg was selected for further booster evaluation. With increasing breakthrough SARS-CoV-2 infections in previously immunized individuals, booster vaccination remains important for addressing waning antibody levels and emerging variants. An ongoing phase 3 study in Laos is further evaluating SW-BIC-213 as a booster against Omicron and other emerging variants of concern in healthy adults who have completed a two-dose series of inactivated COVID-19 vaccines.

## 5. Conclusions

In summary, this study showed that SW-BIC-213 was generally safe and immunogenic in the current study participants, and inoculation with this candidate mRNA vaccine induced measurable humoral immune responses.

## Figures and Tables

**Table 1:** Baseline demographic characteristics of participants in phase 1 and phase 2 trials.

Characteristic	Phase 1 Trial (*n* = 40)			Phase 2 Trial (*n* = 479)				Adults (18–60 Years)				Seniors (>60 Years)			
	25 μg (*n* = 20)	45 μg (*n* = 20)	*p*-Value	Placebo (*n* = 96)	25 μg (*n* = 191)	45 μg (*n* = 192)	*p*-Value	Placebo (*n* = 72)	25 μg (*n* = 143)	45 μg (*n* = 144)	*p*-Value	Placebo (*n* = 24)	25 μg (*n* = 48)	45 μg (*n* = 48)	*p*-Value
Age, mean (SD)	31.3 (10.3)	30.2 (10.0)	0.7337	46.3 (17.2)	47.5 (16.7)	47.3 (17.2)	0.8439	39.0	40.1	40.0	0.7670	68.0	68.6	69.4	0.7327
Age, median	28.5	27.0		46.5	49.0	48.0		39	41	42		65.0	67.5	69.5	
Age, Q1, Q3	23.0, 38.0	23.0, 36.5		32.0, 60.0	32.0, 60.1	33.0, 60.5		28.0, 51.0	28.0, 52.0	29.5, 50.0		61.5, 69.0	63.0, 73.5	62.0, 75.0	
Male	16 (80.0%)	15 (75.0%)	1.0000	62 (64.6%)	92 (48.2%)	108 (56.3%)	0.0265	45 (62.5%)	72 (50.3%)	87 (60.4%)	0.1258	17 (70.8%)	20 (41.7%)	21 (43.8%)	0.0468
Female	4 (20.0%)	5 (25.0%)		34 (35.4%)	99 (51.8%)	84 (43.8%)		27 (37.5%)	71 (49.7%)	57 (39.6%)		7 (29.2%)	28 (58.3%)	27 (56.3%)	
Asian	20 (100.0%)	20 (100.0%)	1.0000	96 (100.0%)	191 (100.0%)	192 (100.0%)	1.0000	72 (100.0%)	143 (100.0%)	144 (100.0%)	1.0000	24 (100.0%)	48 (100.0%)	48 (100.0%)	1.0000
BMI, mean (SD)	21.510 (2.337)	23.175 (3.098)	0.0624	21.452 (3.370)	21.748 (3.475)	21.283 (3.345)	0.4040	21.737 (3.307)	22.096 (3.198)	21.804 (3.314)	0.6649	20.596 (3.483)	20.710 (4.056)	19.718 (2.952)	0.3504
BMI, median	21.719	22.342		21.076	21.406	20.953		21.492	21.567	21.317		19.869	20.425	19.590	
BMI, Q1, Q3	20.1, 23.7	21.5, 23.8		19.1, 23.5	19.5, 23.6	19.2, 23.2		19.3, 23.7	19.8, 23.8	19.9, 23.6		18.5, 21.3	17.7, 23.3	17.9, 21.3	

Data are mean (SD), median, Q1/Q3, or *n* (%). *p*-values were calculated using Student’s *t*-test, Fisher exact test, ANOVA, or chi-square test, as appropriate. BMI, body mass index.

## Data Availability

The original contributions presented in this study are included in the article/Supplementary Materials. Further inquiries can be directed to the corresponding authors. The data that support the findings of this study are available on reasonable request to the authors.

## Supplementary Materials

The following supporting information can be downloaded at: https://www.mdpi.com/article/10.3390/vaccines14080708/s1.

## Abbreviations

LPP	Lipo-Polyplex
LNP	Lipid Nanoparticles
WHO	World Health Organization
Lao PDR	Lao People’s Democratic Republic
DSMB	Data Safety Monitoring Board
SARS	Severe Acute Respiratory Syndrome
MERS	Middle East Respiratory Syndrome
COVID-19	Coronavirus Disease 2019
AIDS	Acquired Immune Deficiency Syndrome
NMPA	National Medical Products Administration
RBD	Receptor Binding Domain
GMT	Geometric Mean Titer
WT	Wild Type
IRT	Interactive Response Technology
FAS	Full Analysis Set
PPS	Per Protocol Set
ITT	Intention-To-Treat
BAU	Binding Antibody Unit
ELISA	Enzyme Linked Immunosorbent Assay
VOC	Variants of Concern
SAE	Serious Adverse Event
ADE	Antibody Dependent Enhancement
VED	Vaccine Enhanced Disease
