# Supplementary material for: Safety and Immunogenicity of SW-BIC-213, a Modified COVID-19 Lipo-Polyplex mRNA Vaccine, in Laotian Healthy Adults Aged 18 Years and Above: A Phase 1/2 Trial
Source: Vaccines (Basel). 2026 Aug 18;14(8):708. doi: 10.3390/vaccines14080708 (PMC13517350; doi:10.3390/vaccines14080708)
Supplement: Supplementary file 1 [file vaccines-14-00708-s001.zip › supplementary figures.pdf]

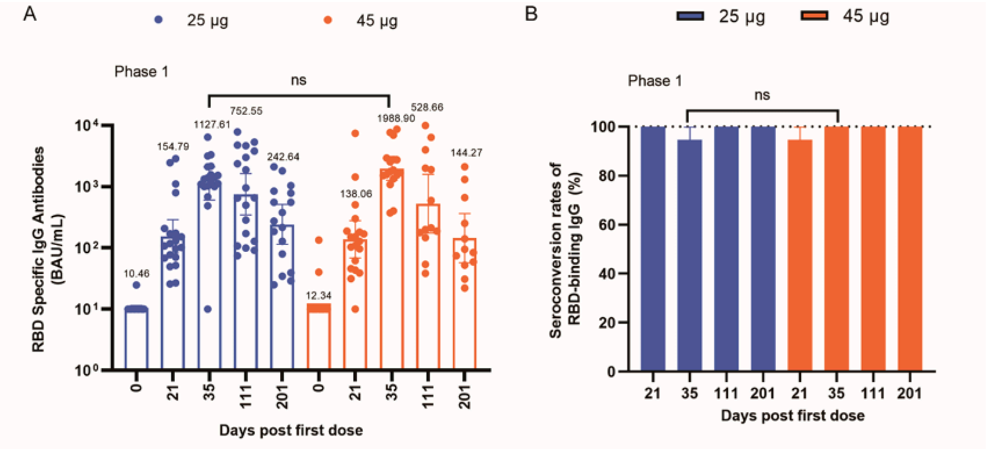

Figure S1. SW-BIC-213 induced RBD binding antibodies in the phase 1 trial. (A) SARS-CoV-2 RBD protein binding antibody titers determined by ELISA. (B) Seroconversion rates of SARS-CoV-2 RBD protein binding antibodies.

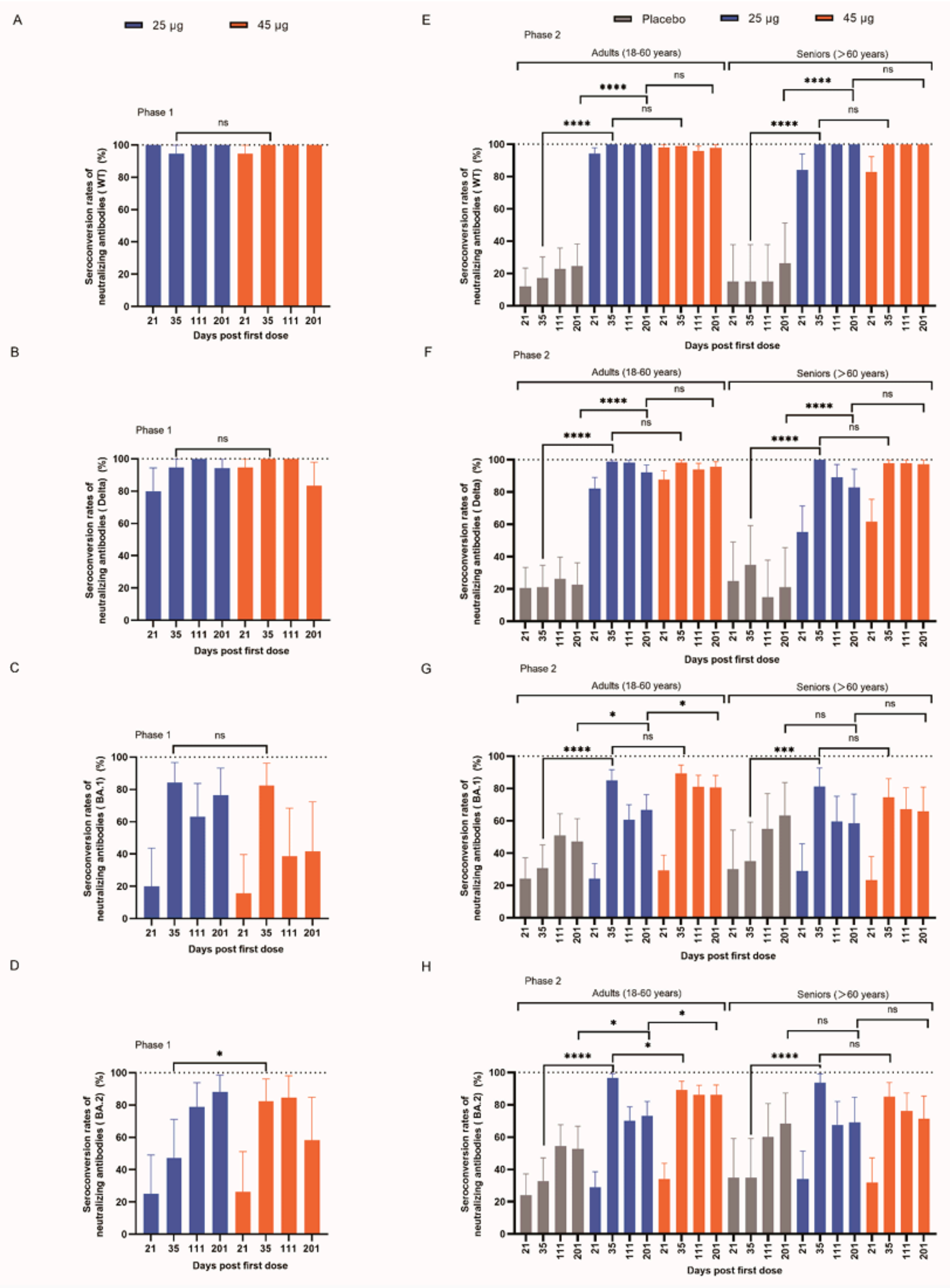

Figure S2. Seroconversion rates of SW-BIC-213-induced pseudovirus neutralizing antibodies in phase 1 and phase 2 trials. (A-D) Seroconversion rates in the phase 1 trial against prototype/WT, Delta, Omicron BA.1, and Omicron BA.2 strains, respectively. (E-H) Seroconversion rates in the phase 2 trial against prototype/WT, Delta, Omicron BA.1, and Omicron BA.2 strains, respectively. Treatment groups and age cohorts are indicated by the axis labels and figure legends.
