# Supplementary material for: Safety and Immunogenicity of SW-BIC-213, a Modified COVID-19 Lipo-Polyplex mRNA Vaccine, in Laotian Healthy Adults Aged 18 Years and Above: A Phase 1/2 Trial
Source: Vaccines (Basel). 2026 Aug 18;14(8):708. doi: 10.3390/vaccines14080708 (PMC13517350; doi:10.3390/vaccines14080708)
Supplement: Supplementary file 1 [file vaccines-14-00708-s001.zip › Supplementary_File_S1_Appendix_and_Supplementary_Methods_vaccines-4439200.pdf]

Supplementary File S1: Appendix and Supplementary Methods

Manuscript ID: vaccines-4439200 | ClinicalTrials.gov: NCT05144139

Safety and immunogenicity of SW-BIC-213, a modified COVID-19 Lipo-Polyplex mRNA vaccine, in Laotian healthy adults aged 18 years and above: a phase 1/2 trial

Note: This supplementary file summarizes manuscript-described eligibility, blinding, and immunogenicity assay procedures corresponding to the revised manuscript.

File S1a. Inclusion and Exclusion Criteria

Inclusion criteria

- Adults eligible for the phase 1 trial were healthy individuals aged 18 to 60 years. Adults eligible for the phase 2 trial were healthy individuals aged 18 years and older, including adult and senior cohorts.
- Participants were considered to be in overall good health based on medical history, physical examination, and laboratory tests at the screening visit.
- Participants were able and willing to provide written informed consent before any study procedure.
- Male and female participants were eligible, provided that they agreed to comply with contraception requirements during the trial, where applicable.
- Participants were willing and able to comply with the vaccination schedule, follow-up visits, blood sampling, safety monitoring, and other protocol-specified procedures.

Exclusion criteria

- Pregnant participants were excluded.
- Participants with a known history of SARS-CoV-2, SARS-CoV-1, or MERS-CoV infection were excluded.
- Participants with COVID-19-like symptoms within 14 days before enrollment were excluded. Symptoms assessed included fever, dry cough, fatigue, nasal congestion, runny nose, sore throat, myalgia, diarrhea, shortness of breath, and dyspnea.
- Participants with serious cardiovascular disease, hypertension, acute respiratory disease, kidney disease, or other clinically significant conditions that could interfere with trial participation or safety evaluation were excluded.
- Participants with clinically significant abnormalities in screening medical history, physical examination, vital signs, or laboratory tests, as judged by investigators, were excluded.
- Participants who were unable to comply with trial procedures or follow-up requirements were excluded.

Known previous SARS-CoV-2 infection was screened by medical history, self-reported infection history, interview, physical examination, and review of COVID-19-related symptoms within 14 days before enrollment. Systematic anti-nucleocapsid antibody testing, PCR screening, and formal baseline seropositivity exclusion were not performed.

File S1b. . Blinding

The phase 1 trial was open-label and dose-escalating; randomization and blinding were not used in phase 1. Eligible participants were assigned in a 1:1 ratio to the 25 microgram or 45 microgram SW-BIC-

213 dose group according to enrollment sequence. Enrollment proceeded stepwise from the 25 microgram dose group to the 45 microgram dose group after review of preliminary 7-day safety data from the lower-dose group.

The phase 2 trial used a randomized, blinded, placebo-controlled, parallel design. Participants were enrolled into adult and senior cohorts. Overall, participants were randomized to 25 micrograms, 45 micrograms, or placebo at an approximate 2:2:1 ratio; within each sequential dose cohort, participants were randomly assigned at a 4:1 ratio to SW-BIC-213 or matched placebo. Randomization was implemented through an interactive response technology system. A randomization statistician generated the randomization list using block randomization (block size 30) in SAS version 9.4, and the list was imported into the interactive response technology system for treatment allocation.

- Authorized unblinded pharmacists prepared SW-BIC-213 or placebo according to treatment allocation.
- Participants, investigators involved in safety assessment, laboratory personnel, immunogenicity assessors, and statisticians remained blinded to treatment allocation during blinded trial conduct and analysis.
- The injected active vaccine volumes were 0.25 mL for the 25 microgram dose and 0.45 mL for the 45 microgram dose. Within each sequential dose cohort, the saline placebo volume was matched to the corresponding active-dose injection volume.
- Emergency unblinding could be performed only when knowledge of the assigned intervention was necessary for participant safety or clinical management.
- A formal blinding-index or allocation-guess-accuracy assessment was not collected.

Supplementary Methods

Sample collection

Blood samples were collected before vaccination, before the second dose, and at 14, 90, and 180 days after the second dose. Serum samples were used for SARS-CoV-2 spike protein-specific IgG assays, RBD-specific IgG assays in phase 1, pseudovirus neutralization assays, and live-virus neutralization assays.

ELISA for SARS-CoV-2 spike- and RBD-specific IgG antibodies

1. Serum samples and assay controls were tested according to laboratory standard operating procedures and previously reported assay methods referenced in the manuscript.
2. Microplates were coated with recombinant SARS-CoV-2 spike protein or receptor-binding-domain antigen, blocked, and incubated with serially diluted serum samples and reference standards.
3. Bound human IgG was detected using an enzyme-conjugated anti-human IgG antibody and chromogenic substrate.
4. Optical density values were measured using a microplate reader, and antibody concentrations or titers were calculated from the standard curve.
5. Results were summarized as geometric mean titers or concentrations and seroconversion rates at protocol-specified time points.

Pseudovirus neutralization assay

6. Pseudovirus neutralizing antibodies were measured against SARS-CoV-2 prototype/wild-type, Delta, Omicron BA.1, and Omicron BA.2 strains.

7. Serially diluted serum samples were mixed with a fixed amount of pseudovirus and incubated to allow antibody-virus interaction.
8. The serum-pseudovirus mixtures were added to susceptible target cells expressing the appropriate viral entry receptor.
9. After incubation, infection was quantified using the assay readout specified in the laboratory standard operating procedure.
10. Neutralizing titers were calculated as the reciprocal serum dilution producing 50% inhibition of pseudovirus infection and were summarized as geometric mean titers and seroconversion rates.

Live-virus neutralization assay

11. Live-virus neutralizing antibodies against the prototype/wild-type SARS-CoV-2 strain were measured using a microneutralization assay under appropriate biosafety containment.
12. Serially diluted serum samples were incubated with live SARS-CoV-2 before addition to susceptible cell monolayers.
13. After incubation, viral infection was assessed according to the laboratory standard operating procedure.
14. Neutralizing titers were reported as reciprocal serum dilution endpoints and summarized as geometric mean titers and seroconversion rates.

Safety assessment methods

Solicited local adverse events within 7 days after vaccination included injection-site pain, pruritus, induration, swelling, and redness. Solicited systemic adverse events within 7 days after vaccination included fever, fatigue, headache, myalgia, chills, arthralgia, nausea, lymphadenopathy, diarrhea, vomiting, and acute allergic reaction. Unsolicited adverse events were collected for 0-21 days after the first dose and 0-28 days after the second dose. Medically attended adverse events, adverse events of special interest, and serious adverse events were monitored according to the protocol.

Adverse event severity was evaluated using the DAIDS Table for Grading the Severity of Adult and Pediatric Adverse Events, the NMPA Guidelines for Adverse Event Classification Standards for Clinical Trials of Preventive Vaccines, and the FDA Toxicity Grading Scale for Healthy Adult and Adolescent Volunteers Enrolled in Preventive Vaccine Clinical Trials, as applicable.
