# Supplementary material for: Safety and Immunogenicity of SW-BIC-213, a Modified COVID-19 Lipo-Polyplex mRNA Vaccine, in Laotian Healthy Adults Aged 18 Years and Above: A Phase 1/2 Trial
Source: Vaccines (Basel). 2026 Aug 18;14(8):708. doi: 10.3390/vaccines14080708 (PMC13517350; doi:10.3390/vaccines14080708)
Supplement: Supplementary file 1 [file vaccines-14-00708-s001.zip › Supplementary_File_S2_Protocol_of_Clinical_Trial_vaccines-4439200.pdf]

Supplementary File S2: Protocol of Clinical Trial

Manuscript ID: vaccines-4439200 | ClinicalTrials.gov: NCT05144139

Safety and immunogenicity of SW-BIC-213, a modified COVID-19 Lipo-Polyplex mRNA vaccine, in Laotian healthy adults aged 18 years and above: a phase 1/2 trial

Document type: Clinical trial protocol summary compiled from the revised manuscript. This file should be cross-checked against the approved full protocol before submission if the Editorial Office requires the original protocol document.

1. Trial Identification

Item	Description
Manuscript ID	vaccines-4439200
Trial registration	NCT05144139
Trial phase	Seamless phase 1/2 clinical trial
Investigational product	SW-BIC-213, a modified COVID-19 Lipo-Polyplex mRNA vaccine
Study population	Healthy adults in Lao PDR
Study sites	Clinical sites in Vientiane and Savannakhet, Lao PDR, as described in the revised manuscript

2. Objectives

- To evaluate the safety and tolerability of two intramuscular doses of SW-BIC-213 administered 21 days apart.
- To evaluate humoral immunogenicity after vaccination, including spike-specific IgG antibodies and neutralizing antibodies against SARS-CoV-2 strains.
- To compare immune responses between the 25 microgram and 45 microgram dose levels and placebo, where applicable.

3. Trial Design

This was a seamless phase 1/2 clinical trial conducted in healthy adults in Lao PDR. Phase 1 used an open-label, dose-escalation design. Phase 2 used a randomized, blinded, placebo-controlled, parallel design. The trial was conducted at clinical sites in Vientiane and Savannakhet, Lao PDR, as described in the revised manuscript.

Trial part	Design	Population	Intervention
Phase 1	Open-label dose escalation	Healthy adults aged 18-60 years	SW-BIC-213 25 micrograms or 45 micrograms, two doses 21 days apart
Phase 2	Randomized, blinded, placebo-controlled, parallel trial	Healthy adults aged >=18 years, including adult and senior cohorts	SW-BIC-213 25 micrograms, SW-BIC-213 45 micrograms, or saline placebo; overall allocation approximately 2:2:1, with 4:1 active-to-placebo assignment within each sequential dose cohort; two doses 21 days apart

4. Eligibility

Participants were screened using medical history, interview, physical examination, vital signs, and laboratory tests. The inclusion and exclusion criteria are summarized in Additional File 1, Appendix 1.

5. Investigational Product and Administration

- SW-BIC-213 encodes a modified full-length spike protein based on the ancestral Wuhan-Hu-1 isolate of SARS-CoV-2.
- The encoded spike contains K986P/V987P prefusion-stabilizing substitutions, a 682-QSAQ-685 furin-cleavage-site substitution, and D614G.
- The vaccine was formulated using the Lipo-Polyplex platform and was developed and manufactured by StemiRNA Therapeutics Co., Ltd., Shanghai, China, in accordance with good manufacturing practice guidelines.
- Frozen vaccine vials were thawed under controlled conditions according to the protocol and manufacturer handling instructions, kept at 2-8 degrees C after thawing, and prepared during the same vaccination session.
- The injected active vaccine volumes were 0.25 mL for the 25 microgram dose and 0.45 mL for the 45 microgram dose. Placebo was a preservative-free 0.9% NaCl saline solution matched by injection volume within the corresponding dose cohort.
- Vaccinations were administered intramuscularly into the upper-arm deltoid muscle 21 days apart.
- Participants were observed on site for 30 minutes after each vaccination.

6. Randomization and Blinding

Phase 1 was open-label and participants were assigned to dose groups according to enrollment sequence. In phase 2, participants were randomized overall to 25 micrograms, 45 micrograms, or placebo at an approximate 2:2:1 ratio; within each sequential dose cohort, participants were randomly assigned at a 4:1 ratio to SW-BIC-213 or matched placebo. Randomization was performed through an interactive response technology system using a statistician-generated block randomization list (block size 30) prepared in SAS version 9.4. Authorized unblinded pharmacists prepared assigned products; participants and blinded study personnel remained masked to treatment allocation. Additional details are provided in Additional File 1, Appendix 2.

7. Safety Assessments

- Participants were monitored for immediate adverse reactions during the 30-minute post-vaccination observation period.
- Solicited local and systemic adverse events were collected for 7 days after each vaccination.
- Unsolicited adverse events were collected for 0-21 days after the first dose and 0-28 days after the second dose.
- Medically attended adverse events, adverse events of special interest, and serious adverse events were monitored according to the protocol.
- In phase 1, blood and urine samples were collected before and 4 days after each dose for hematology and chemistry laboratory tests.
- Body temperature was measured consistently at the axillary site during safety follow-up.

8. Immunogenicity Assessments

- Blood samples were collected before each vaccination and at 14, 90, and 180 days after the second dose.
- SARS-CoV-2 spike protein-specific IgG antibodies were measured by ELISA.
- RBD-specific IgG antibodies were assessed in the phase 1 trial as an exploratory endpoint.
- Pseudovirus neutralizing antibodies were measured against prototype/wild-type, Delta, Omicron BA.1, and Omicron BA.2 strains.
- Live-virus neutralizing antibodies against the prototype/wild-type strain were measured using a live-virus neutralization assay (microneutralization assay).

9. Endpoints

Endpoint category	Endpoint
Primary safety endpoints	Incidence and severity of solicited local/systemic adverse reactions, unsolicited adverse events, medically attended adverse events, adverse events of special interest, and serious adverse events during protocol-specified observation periods
Primary immunogenicity endpoints	Seroconversion rates and geometric mean titers of SARS-CoV-2 spike-specific IgG antibodies and neutralizing antibodies 14 days after the second dose
Exploratory endpoints	RBD-specific IgG antibodies in phase 1 and persistence of humoral immune responses at 90 and 180 days after the second dose

10. Sample Size

In phase 1, the probability of observing a specific adverse event with an incidence of 8% at least once in 20 participants in each dose group was 81.1%; 41 participants were enrolled. In phase 2, the sample size was based on assumed seroconversion rates of 80% in vaccine groups and 10% in placebo groups, a one-sided alpha of 0.025, and 33% dropout. The planned sample size provided at least 99% power; 480 participants were enrolled. In the revised manuscript, 94 individuals were screened in phase 1 and 863 individuals were screened in phase 2; 40 phase 1 participants and 479 phase 2 participants received the first vaccination.

11. Analysis Sets and Statistical Analysis

- The safety analysis set included all participants who received at least one dose of investigational product.
- The full analysis set included all participants who received at least one vaccination and had valid pre-vaccination antibody results.
- The per-protocol set included participants who met eligibility criteria, completed the protocol-defined vaccination course, and had valid pre- and post-vaccination immunogenicity data.
- Safety endpoints were summarized as percentages of participants with adverse events or serious adverse events.
- Seroconversion rates and 95% confidence intervals were calculated using the Clopper-Pearson method.
- Fisher exact tests or chi-square tests were used for categorical comparisons. Geometric mean titers were compared after log transformation using Student t-tests; when ANOVA indicated an overall difference among three or more groups, prespecified pairwise comparisons were performed.
- No formal multiplicity adjustment was applied; p values were interpreted as descriptive for multiple comparisons.

12. Ethics and Oversight

The trial received approval from the Ethics Committee and Food & Drug Department of the Ministry of Health, Lao PDR (certificate No. 7745). The protocol and each amendment were reviewed by the Independent Ethics Committee of the Lao People's Democratic Republic Ministry of Health National Ethics Committee for Health Research. All participants provided written informed consent before participation. The study adhered to the Declaration of Helsinki and Good Clinical Practice Guidelines. Safety assessment, including the need for study suspension or termination, was conducted by an independent Data Safety Monitoring Board.
